# Description of two fatal cases of melioidosis in Mexican children with acute pneumonia: case report

**DOI:** 10.1186/s12879-021-05910-5

**Published:** 2021-02-23

**Authors:** Gerardo Alvarez-Hernandez, Denica Cruz-Loustaunau, J. Antonio Ibarra, Adela Rascon-Alcantar, Jesús Contreras-Soto, Georgina Meza-Radilla, Alfredo G. Torres, Paulina Estrada-de los Santos

**Affiliations:** 1 Department of Medicine and Health Sciences, University of Sonora, Hermosillo, Sonora Mexico; 2 Ministry of Health of Sonora, Hermosillo, Sonora Mexico; 3 Instituto Politécnico Nacional, Escuela Nacional de Ciencias Biológicas, Prol. Carpio y Plan de Ayala s/n, Col. Santo Tomás, Alcaldía Miguel Hidalgo. C.P., 11340 Mexico City, Mexico; 4 Sonora Children’s Hospital, Hermosillo, Sonora Mexico; 5 University of Texas Medical Branch, Galveston, TX USA

**Keywords:** Melioidosis, *Burkholderia pseudomallei*, Fatal outcome, Case report

## Abstract

**Background:**

Melioidosis is an infectious disease caused by *Burkholderia pseudomallei*. In Mexico, the disease is rarely diagnosed in humans and there is no evidence of simultaneous environmental isolation of the pathogen. Here, we describe clinical profiles of fatal cases of melioidosis in two children, in a region without history of that disease.

**Case presentation:**

About 48 h before onset of symptoms, patients swam in a natural body of water, and thereafter they rapidly developed fatal septicemic illness. Upon necropsy, samples from liver, spleen, lung, cerebrospinal fluid, and bronchial aspirate tissues contained *Burkholderia pseudomallei*. Environmental samples collected from the locations where the children swam also contained *B. pseudomallei*. All the clinical and environmental strains showed the same BOX-PCR pattern, suggesting that infection originated from the area where the patients were swimming.

**Conclusions:**

The identification of *B. pseudomallei* confirmed that melioidosis disease exists in Sonora, Mexico. The presence of *B. pseudomallei* in the environment may suggest endemicity of the pathogen in the region. This study highlights the importance of strengthening laboratory capacity to prevent and control future melioidosis cases.

**Supplementary Information:**

The online version contains supplementary material available at 10.1186/s12879-021-05910-5.

## Background

Melioidosis is a disease caused by *Burkholderia pseudomallei,* this bacterial species is frequently found in soil and fresh surface water [1]. Humans and animals become infected through abrasions in the skin, inoculation through mucosal membranes, inhalation of aerosols, or ingestion of contaminated water [1, 2]. This disease is a well-known public health problem in Northern Australia and Southeast Asia [3]. Melioidosis is presumed to be present but underreported in several developing tropical countries around the world, where a mixture of social, biological and health-care related factors converge to favor the presence of *B. pseudomallei* in the environment and the occurrence of human cases and deaths [4]. In the Americas, melioidosis has been declared endemic in several countries, including Brazil, Puerto Rico, and Colombia [5]. This bacterium has been isolated from children with pneumonia in Mexico City, but melioidosis disease has not been commonly diagnosed [6]. Historically, Mexico is considered a country with sporadic melioidosis cases but with no environmental evidence of the bacterium [7]; however, there are documented cases of disease in Mexican patients with no history of traveling overseas or to established endemic areas [8, 9].

Melioidosis can cause a wide range of acute, chronic, or latent clinical manifestations, although most infected people can control and eliminate the bacteria and have only subclinical symptoms [10]. While in some regions of the world, melioidosis causes a high lethality, ranging between 20 to 50%, the disease can be prevented and, if treated promptly, will respond to antibiotic treatment [11]. Because disease can persist, some cases may resemble tuberculosis or other chronic infections [12]. Clinical manifestations have been grouped into four classes: a) acute fulminant septicemia; b) subacute disease; c) chronic disease; and d) subclinical disease [13, 14]. Thus, clinicians, laboratorians, and other health professionals face a challenge to recognize the infection in a timely manner, confirm the diagnosis, and implement measures to prevent and control community cases [15]. Here, we describe two fatal cases of melioidosis occurring in two siblings from a northwestern rural area in Mexico, with the goal of contributing to a better understanding of the disease in a region previously not known to be endemic for melioidosis.

## Case presentation

**Fig. 1:**
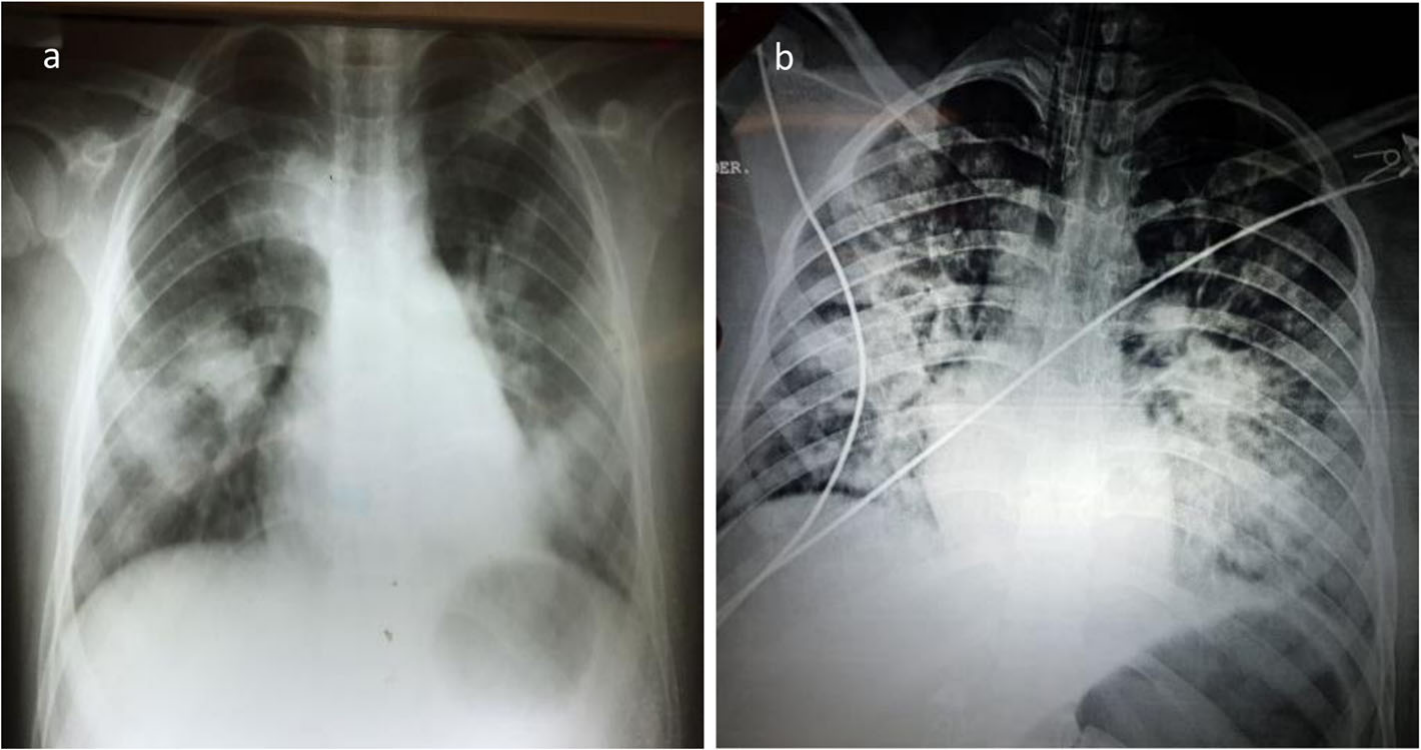
Chest X-ray upon hospital admission of children infected with melioidosis*.* a, 12-year old male patient showing basal radio-opacities in both lungs. b, 16-year old female patient showing disseminated bilateral micronodular infiltrate in both lungs. Both patients received the antibiotics ceftazidime (2 g IV e/8 h), vancomycin (10 mg/Kg IV, e/6 h) and fluconazole (10 mg/Kg IV, e/24 h)

**Fig. 2:**
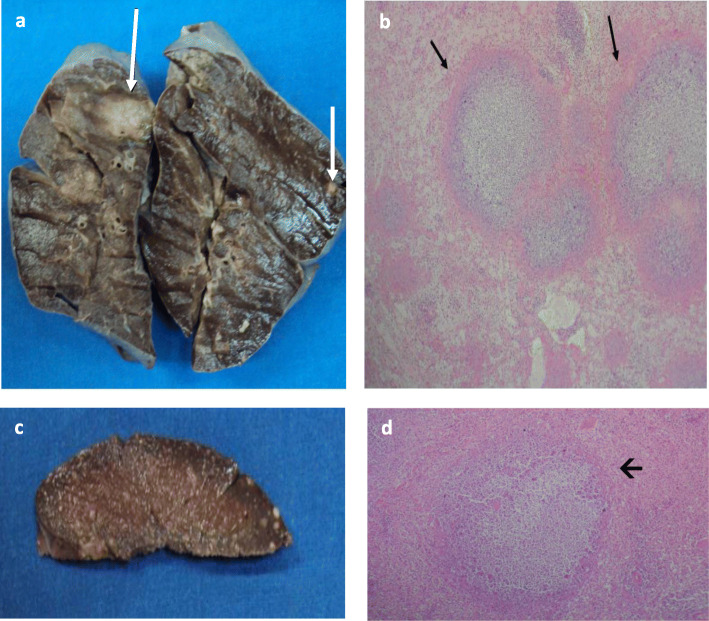
Macroscopic and microscopic images of lungs and spleen from a fatal case of melioidosis. a, macroscopic aspect of the lungs with several abscesses. b, microscopic image of the lung showing the multiple abscesses from the male patient. c, macroscopic aspect of the spleen with splenomegaly and many abscesses. d, microscopic image of the spleen with destruction of the splenic parenchyma in the central territory of the cavity. The arrows indicate the abscesses

On September 2, 2018, a 12-year-old male was admitted in a local community hospital in the town of Huasabas, Sonora, Mexico (Fig. S1). On September 5, 2018, his 16-year-old sister was also admitted to the hospital. Both presented with intense headache, fever, abdominal and chest pain, nausea, and diarrhea. Non-specific medication was prescribed in addition to rest at home. Two days before the admission of the male patient, the brother and sister went swimming in “El Cajón del Chotaqui”, a natural pool of rainwater close to the Huasabas community (Fig. S2). The patients and relatives did not travel out of the town during the previous month and no history of previous diseases was documented in either sibling. After 24 h of disease progression, there was no amelioration of symptoms, therefore both patients were referred to a pediatric hospital in the state capital city of Sonora. Both children presented with severe leukopenia, respiratory distress syndrome and septic shock (Table 1). Chest X-rays showed radio-opacities clinically compatible with rapidly evolving pneumonia (Fig. 1). The patients received a single dose of intravenous therapy with ceftazidime (2 g), vancomycin (10 mg/Kg) and fluconazole (10 mg/Kg) as medications of choice, as well as respiratory and hemodynamic support, which included orotracheal intubation with assisted mechanical ventilation; vasopressor amines were administered due to hemodynamic instability. Nonetheless, they continued with poor evolution, until irreversible cardiorespiratory arrest and death 7 h after admission. No causative agent was suspected at this stage. Upon necropsy, multiple abscesses were found in the larynx, trachea, liver, spleen, lungs, lymph nodes and bone marrow (Fig. 2). Hemophagocytic syndrome, septic shock, bilateral pulmonary hemorrhage, acute tubular necrosis, gastroenterocolitis and chronic cystitis, as well as cerebral edema were documented. Samples from liver, spleen, lung, cerebrospinal fluid, and bronchial aspirate tissues were collected. The samples were inoculated in blood agar and MacConkey medium. A single colony morphology microorganism was preferentially isolated and identified with the VITEK2 System. The results identified the microorganisms HLCR2, HLCR3 and HLCR7 as *B. pseudomallei*, the causative agent of melioidosis.

**Table 1:** Laboratory findings in two fatal cases of melioidosis

Parameters	Case 1	Case 2	Reference values
Hemoglobin (HGB)	9.7 g/dL	10.30 g/dL	12.2–18.1 g/dL
Leukocytes (WBC)	900 μL	750 μL	4600–10,200 μL
Neutrophils (NEUT)	450 μL	280 μL	2800–5200 μL
Lymphocytes (LYM)	420 μL	230 μL	1400–3150 μL
Platelets (PLT)	69,000 μL	93,000 μL	150,000–500,000 μL
Aspartate aminotransferase (AST)	108 μL	121 U/L	0–32 U/L
Alanine aminotransferase (ALT)	39 μL	47 U/L	0–33 U/L
Total bilirubin	0.5 mg/dL	0.8 mg/dL	0.0–12 mg/dL
Serum creatinine	1.1 mg/dL	1.6 mg/dL	0.52–1.04 mg/dL
Blood urea nitrogen	61.3 mg/dL	55 mg/dL	15.0–36.4 mg/dL
Procalcitonin	89.3 ng/mL	23.4 ng/mL	< 0.5 ng/mL
LDH	1125 U/L	909 U/L	240–480 U/L

**Fig. 3:**
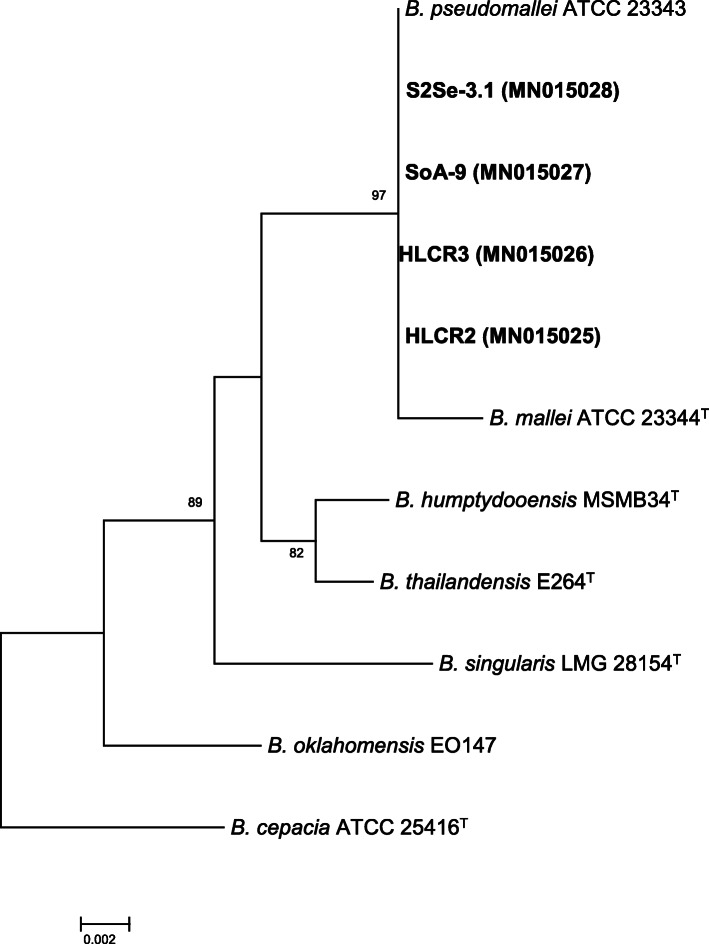
Phylogenetic relationship among *Burkholderia pseudomallei* group species based in the analysis of 16S rRNA gene sequence by maximum likelihood under the model GTR + G. In bold are the strains analyzed in this study. In parenthesis are the accession number in the GenBank database. The bar means the expected differences among sequences

**Fig. 4:**
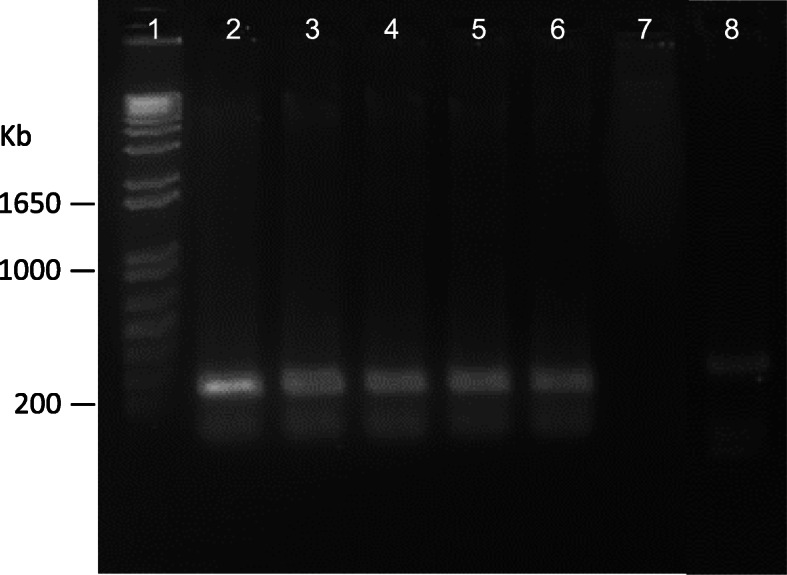
BOX-PCR from *Burkholderia pseudomallei* strains. Lanes: 1, 1 Kb molecular marker; 2, HLCR2; 3, HLCR3; 4, HLCR7; 5, SoA-9; 6, S2Se-3.1; 7, Negative control; 8, Positive control *Burkholderia contaminans* UNL15–3

Environmental samples were collected from “El Cajón del Chotaqui”. Soil samples (100 g) were collected at depth of 20 cm and placed in plastic bags. Sediment samples were obtained at a depth of 20–30 cm in the riverbank or at small ponds in “El Cajón del Chotaqui” and stored in 50 ml sterile tubes. The water samples (30–40 ml) were taken from the river surface about half a meter from the riverbank in 50 ml sterile tubes. The soil was resuspended in sterile distilled water and 15 μl from the three environmental samples (resuspended soil, sediment, and water) were streaked on an Ashdown medium plates [16]. All plates were incubated for 3 days at 37 °C. Colonies having the typical *B. pseudomallei* morphology were selected and the DNA was obtained from isolates SoA-1 and S2Se-3.1 [17]. The 16S rRNA from clinical and environmental isolates was amplified with primers 27f and 1492r [18] and sequenced at Macrogen (https://dna.macrogen.com/). The resulting sequences were assembled with the ChromasPro software (Technelysium Pty Ltd) and compared in the EzBiocloud database [19]. This analysis showed the organism to be *B. pseudomallei* (99.93% similarity). The sequences were deposited at NCBI with the accession numbers MN015028 (S2Se-3.1), MN015027 (SoA-9), MN015026 (HLCR3) and HLCR2 (MN015025). An alignment of *Burkholderia* species was performed with MUSCLE (EMBL-EBI) and a phylogenetic tree was obtained using maximum likelihood under the model GTR + G with the program PhyML 3.0. The phylogenetic analysis showed that the strains were associated with the species *B. pseudomallei* (Fig. 3). A BOX PCR analysis was performed using the BOX element (BOXA1) with the BOXA1R primer [20]. The BOX element belongs to a family of repetitive DNA sequences dispersed throughout the genome of diverse bacterial species which may indicate structure and evolution of bacterial genomes. In bacterial taxonomy, BOX elements can indicate clonality or can be used to differentiate strains from a single species. In this analysis, the isolated strains displayed the exact same pattern, which suggests that the strains belong to the same clonal group (Fig. 4).

## Discussion and conclusion

Melioidosis is an endemic infectious disease from tropical regions of the world, and recent increases in cases have been reported in the Americas [21]. Improvements in diagnostic capacity have been linked to a better identification of the pathogen, although a remaining challenge to overcome is underreporting, which could be associated with the lack of clinical knowledge of the disease by physicians [5, 10]. An early recognition of clinical characteristics is key to initiating specific medical treatment, and to establishing preventive and control measures at the community level.

Clinical diagnosis of melioidosis is difficult for physicians, particularly in regions where the pathogen is barely known, and because of its clinical similarity with other infectious pathologies, such as tuberculosis and pulmonary coccidioidomycosis. Early recognition of the disease is key to prevent the development of fulminant sepsis, severe pneumonia, and multiple abscesses [2, 13], a group of clinical manifestations that were observed in the two fatal cases described in this study. Both siblings developed the septicemic illness, involving the lung parenchyma and pleural cavity, resulting in multiple pulmonary abscesses, septic shock with respiratory distress and pneumonia, which is the most common manifestation of acute melioidosis [22, 23].

Known predictors of mortality in septicemic melioidosis were also documented in the two siblings: pulmonary presentation, blood urea nitrogen (BUN), and high white blood cell counts [24]. Interestingly, occurrence of pancytopenia was also observed in the siblings, in addition to the presence of splenomegaly, hepatomegaly with hemophagocytosis of all blood cell lines in bone marrow, liver, and lymph nodes, meeting the clinical and pathological criteria of hemophagocytic syndrome [25]. Further, hyperplasia of macrophages, with hemophagocytosis of all blood cell lines (i.e. erythrocytes, leukocytes, platelets) was observed, which has already been documented in another fatal case [25]. Our findings, combined with previous reports, may guide clinicians to suspect melioidosis in patients with fever, pneumonia, alterations in white blood cells (WBC), thrombocytopenia, and increase of BUN. This is important especially in this region of Mexico where the presence of *B. pseudomallei* has now been reported in clinical cases and isolated from the environment.

On the other hand, it is critical that clinicians consider that *B. pseudomallei* can be misidentified as a culture contaminant or another species, particularly *Pseudomonas* spp., *Bacillus* spp. or *Burkholderia cepacia*, and which often leads to an incorrect diagnosis [15]. It is paramount to consider that *B. pseudomallei* is never part of the normal microbiota and its finding should be considered as a confirmatory evidence of infection and as causative agent of melioidosis [10]. Further, the disease may be easily confirmed when clinically suspected, especially in patients who present fever and have a history of travel to endemic regions or with a history of recreational or occupational environmental exposure [26].

A thorough interrogation linking epidemiological clues to clinical and laboratory findings may guide not only the diagnosis and medical care of patients, but the immediate public health response to identify or prevent further melioidosis cases. In regions where the disease is not frequently diagnosed, clinicians should routinely incorporate questions about environmental conditions (e.g. humidity > 80%, temperature between 68 and 86 °F, and rainfall), as well as potential exposures to contaminated sources. Although melioidosis is more frequent in adults, children and adolescents may have a high risk of being infected by *B. pseudomallei*, due to the likelihood of environmental exposure, either by inhalation of the pathogen or through wounds while swimming in contaminated water [27, 28]. This was probably the case with these two children, because *B. pseudomallei* was found in water and sediment in the area where the children used swam and neither had diabetes as a risk factor.

The environmental and clinical strains showed the same BOX-PCR pattern. The description of the two fatal cases may be of relevance because to the best of our knowledge, there is no documented evidence about the simultaneous presence of *B. pseudomallei* in human cases and environmental samples from Mexico. Recently, several reports [7–9] described the occurrence of melioidosis in different regions of Mexico, but none of them have been able to confirm the environmental presence of the bacteria. Of note, *B. pseudomallei* has been recently reported in children with pneumonia in Mexico City, but melioidosis disease was not diagnosed [6].

Based on the cumulative evidence, Mexico should be considered as an endemic region for *B. pseudomallei*, which may help guide clinicians and public health workers during diagnosis of the disease, and to establish medical treatment and preventive measures as soon as possible. This is of particular importance because Mexico has been found to be the country with the highest predicted incidence of melioidosis in North America, with 550 cases per 100,000 population each year [7]. Several factors such as limited capacity for diagnosis, medical care, and epidemiological surveillance may explain the underreported incidence of the disease [3]. The environmental presence of *B. pseudomallei* might confirm the endemicity of the pathogen in this region. We hope this report will increase awareness about the occurrence of melioidosis in previously unrecognized regions, and prompt action to improve physicians’ training, to strengthen laboratory capacity and to initiate epidemiologic responses that will help prevent and control future cases.

## Supplementary Information

**Additional file 1 Fig. S1**. Localization of Huásabas, Sonora, Mexico. Map shows the Mexican state of Sonora, and the inset map in the upper left corner shows the state’s geographical location in the country. The number indicates the approximate location of Huásabas in Sonora. The map was modified from https://www.inegi.org.mx/app/mapas/ and used under the free use terms by INEGI, Mexico. INEGI, Instituto Nacional de Estadística Geografía e Informática.

**Additional file 2 Fig. S2**. Images of Cajón del Chotaqui in Huásabas, Sonora, México. Pictures were taken during the sampling of water, sediment and soil for *Burkholderia pseudomallei* isolation by the authors of this study.

## Data Availability

The 16S rRNA gene sequence are available at NCBI database with the accession numbers MN015028 (S2Se-3.1), MN015027 (SoA-9), MN015026 (HLCR3) and HLCR2 (MN015025).

## Abbreviations

BUN: blood urea nitrogen
WBC: white blood cells
BOX-PCR: BOX-A1R-based repetitive extragenic palindromic-PCR
