# Supplementary material for: Description of two fatal cases of melioidosis in Mexican children with acute pneumonia: case report
Source: BMC Infect Dis. 2021 Feb 23;21:204. doi: 10.1186/s12879-021-05910-5 (PMC7903701; doi:10.1186/s12879-021-05910-5)

Figure S1. Localization of Huásabas, Sonora, Mexico. Map shows the Mexican state of Sonora, and the inset map in the upper left corner shows the state's geographical location in the country. The number indicates the approximate location of Huásabas in Sonora. The map was modified from https://www.inegi.org.mx/app/mapas/ and used under the free use terms by INEGI, Mexico. INEGI, Instituto Nacional de Estadística Geografía e Informática.

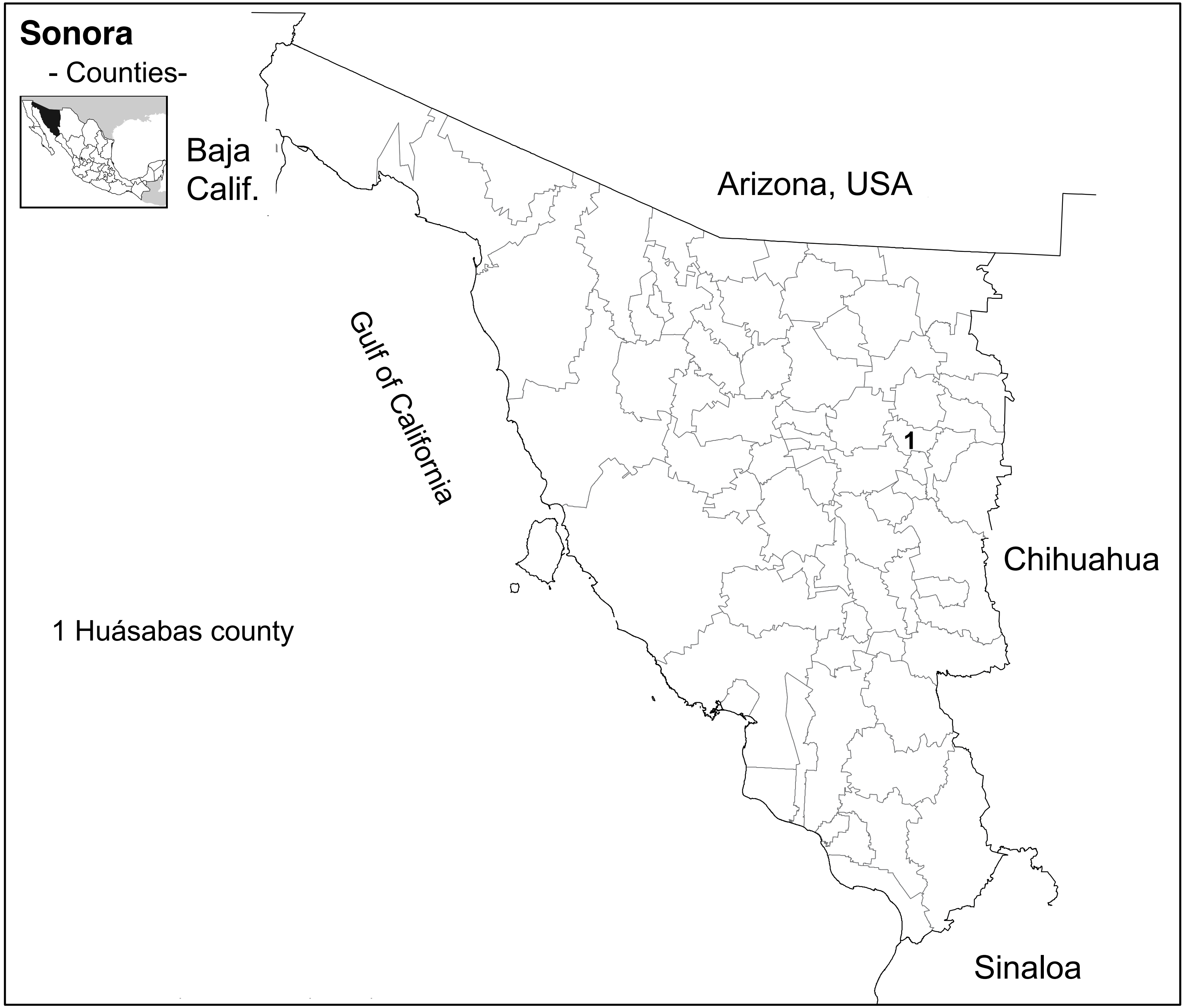

Supplement: Supplementary file 1 — Additional file 1 Fig. S1. Localization of Huásabas, Sonora, Mexico. Map shows the Mexican state of Sonora, and the inset map in the upper left corner shows the state’s geographical location in the country. The number indicates the approximate location of Huásabas in Sonora. The map was modified from https://www.inegi.org.mx/app/mapas/ and used under the free use terms by INEGI, Mexico. INEGI, Instituto Nacional de Estadística Geografía e Informática. [file 12879_2021_5910_MOESM1_ESM.pdf]
