# Supplementary material for: Description of two fatal cases of melioidosis in Mexican children with acute pneumonia: case report
Source: BMC Infect Dis. 2021 Feb 23;21:204. doi: 10.1186/s12879-021-05910-5 (PMC7903701; doi:10.1186/s12879-021-05910-5)
Supplement: Supplementary file 2 — Additional file 2 Fig. S2. Images of Cajón del Chotaqui in Huásabas, Sonora, México. Pictures were taken during the sampling of water, sediment and soil for Burkholderia pseudomallei isolation by the authors of this study. [file 12879_2021_5910_MOESM2_ESM.pdf]

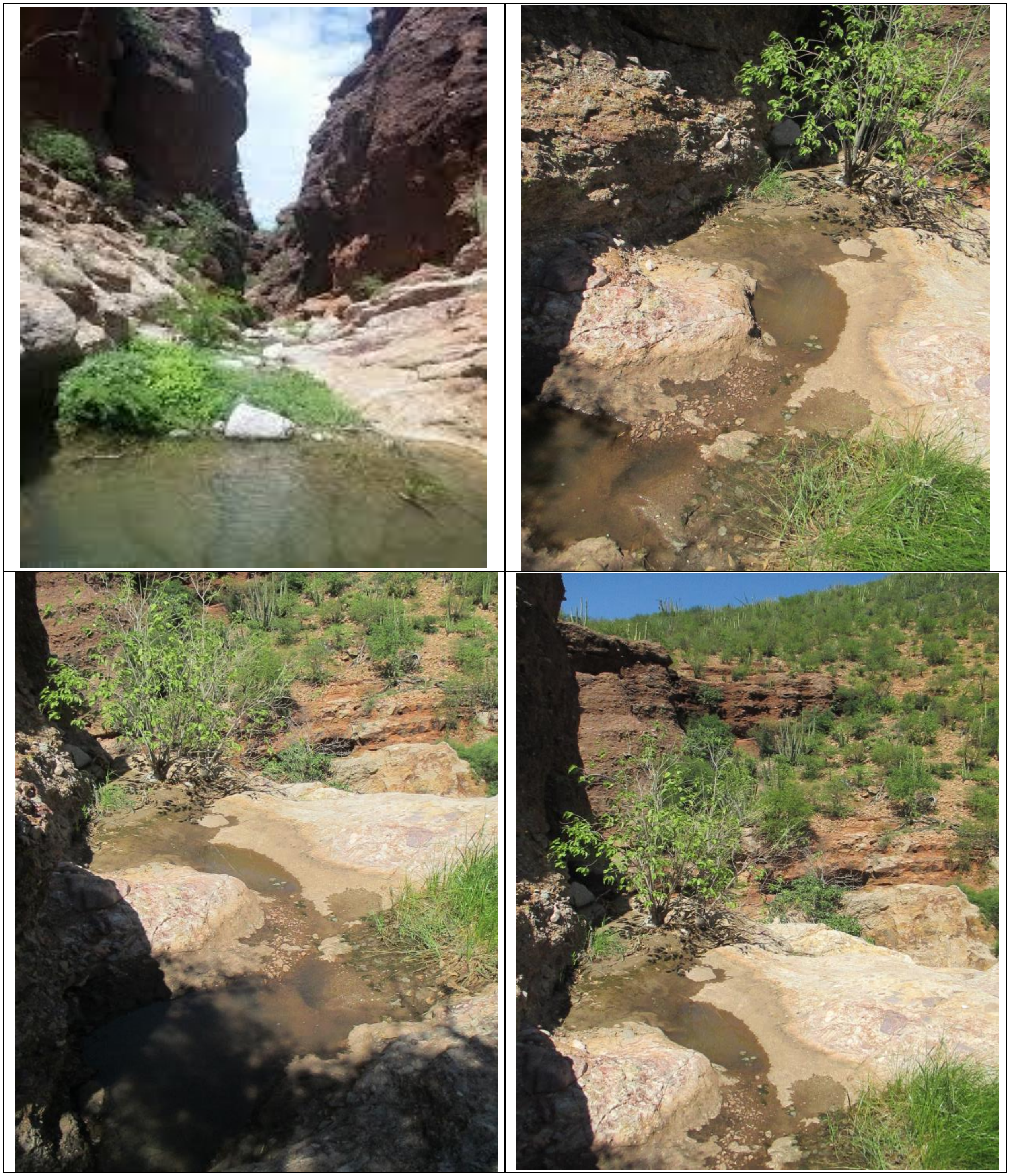

Figure S2. Images of Cajón del Chotaqui in Huásabas, Sonora, México. Pictures were taken during the sampling of water, sediment and soil for *Burkholderia pseudomallei* isolation by the authors of this study.
